# Association of Socioeconomic Status Assessed by Areal Deprivation With Cancer Incidence and Detection by Screening in Miyagi, Japan Between 2005 and 2010

**DOI:** 10.2188/jea.JE20220066

**Published:** 2023-10-05

**Authors:** Noriko Kaneko, Yoshikazu Nishino, Yuri Ito, Tomoki Nakaya, Seiki Kanemura

**Affiliations:** 1 Department of Epidemiology and Public Health, Kanazawa Medical University, Ishikawa, Japan; 2 Faculty of Nursing, Ishikawa Prefectural Nursing University, Ishikawa, Japan; 3 Department of Medical Statistics, Research and Development Center, Osaka Medical and Pharmaceutical University, Osaka, Japan; 4 Graduate School of Environmental Studies, Tohoku University, Sendai, Japan; 5 Division of Cancer Epidemiology and Prevention, Miyagi Cancer Center Research Institute, Miyagi, Japan

**Keywords:** socioeconomic status, areal deprivation index, cancer incidence, cancer stage, screening

## Abstract

**Background:**

Previous studies have shown that socioeconomic factors are associated with cancer incidence and stage at diagnosis; however, relevant findings in Japan are limited. We examined the association between socioeconomic status and cancer incidence, stage at diagnosis, and detection status by screening, as assessed using the areal deprivation index (ADI), in population-based cancer registry data.

**Methods:**

A total of 79,816 cases, including stomach, colorectal, lung, female breast, and cervical cancer diagnosed in Miyagi Prefecture between 2005 and 2010, were analyzed. After calculating the ADI at the place of residence in each case, we examined the association between quintiles of ADI and age-adjusted incidence rates of all stages and advanced stages by sex and site using Poisson regression analysis. The association between the ADI and the proportion of screen-detected cancers was also examined using logistic regression analysis.

**Results:**

The age-adjusted incidence rates of all sites and lung cancer in men and lung cancer and cervical cancer in women tended to increase significantly in areas with a higher ADI. The age-adjusted incidence rates of advanced-stage cancers were significantly higher for all sites and lung cancer in both sexes, and for stomach and colorectal cancer in men. The proportion of screen-detected cancer tended to be significantly lower in areas with a higher ADI for stomach and colorectal cancer in men.

**Conclusion:**

Our results indicate that socioeconomic disparities may affect cancer incidence and early diagnosis in Japan. These results suggest the importance of cancer control measures targeting people with low socioeconomic status in Japan.

## INTRODUCTION

Disparities in cancer incidence exist between regions and identifying the determinants behind each region is useful in resolving these disparities. Previous studies have reported that cancer incidence is associated with the socioeconomic status of the region.^1^^,^^2^ In particular, the areal deprivation index (ADI), calculated from available public statistics, has been used in many studies to assess the socioeconomic status of the community, as it is difficult to directly collect information, including educational history and income, from each resident. The ADI is a synthetic indicator that measures the geographical concentration of multifaceted physical and social poverty, crucially indicating poverty and deprivation levels in the region.^3^ Deprived areas are inhabited by more socio-economically disadvantaged people, who tend to suffer from health disadvantages related to their lower socio-economic status. In addition, living in deprived areas may have a negative impact on the health status of residents due to inadequate living conditions independent of individual socio-economic factors.^4^^–^^7^

The association between ADI and cancer incidence, such as a high incidence of stomach, lung, and cervical cancers in deprived areas, has been previously reported.^8^^–^^10^ This may be due to risk factors associated with socioeconomic status, including smoking and human papillomavirus (HPV) infection in cervical cancer.^6^^,^^9^^,^^11^ On the other hand, breast cancer may be associated with a higher incidence in wealthy areas,^8^^,^^9^^,^^12^ of which a higher proportion of nulliparous women with high socioeconomic status may have an increased risk of breast cancer.^13^

Furthermore, in addition to a high incidence of cancer in deprived areas, advanced cancers and late-stage diseases at the time of diagnosis are also related.^14^^,^^15^ These findings may be due to the low screening rate of cancer and the high proportion of people with delayed consultation behavior even after the onset of symptoms.

Most studies examining the association between ADI and cancer incidence have been conducted in Western countries, and only a few have been conducted in Japan.^16^^,^^17^ Therefore, we examined the relationships between socioeconomic status evaluated using the ADI of small subregions (*chocho-aza* units, average population: 3,000) calculated from the census data and cancer incidence, advanced cancer incidence, and cancer detection by screening using the population-based cancer registry data of Miyagi Prefecture. In this study, we focused on the types of cancers targeted for cancer screening. This is because the cancer screening uptake rate is low in Japan, and it is necessary to accumulate evidence to improve this rate.

## METHODS

### Patients

**Figure 1.**
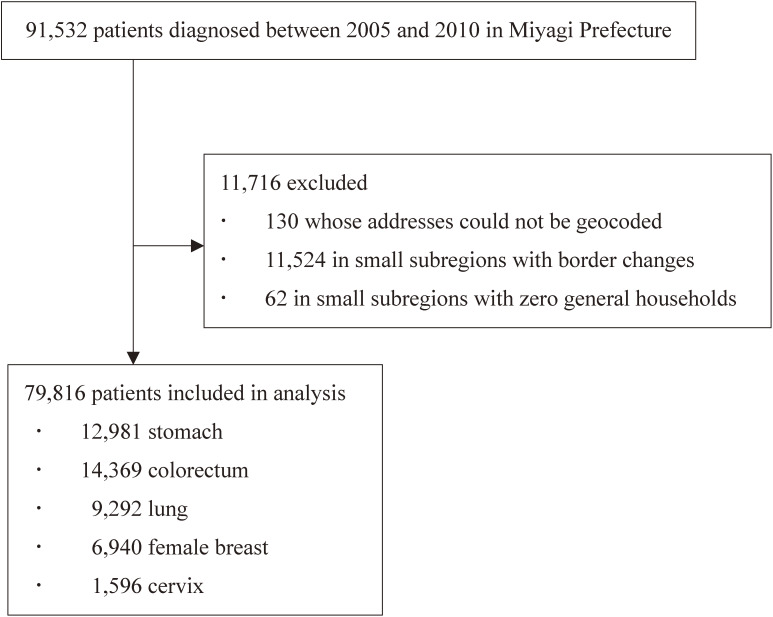
Flowchart of eligible patients

This study was conducted using information from patients diagnosed between 2005 and 2010 from the Miyagi Prefectural Cancer Registry. From a total of 91,532 cases for all cancer sites including carcinoma in situ, 130 cases whose addresses could not be geocoded from the data of the cancer registry, 11,524 cases in areas with border changes in the 2005 and 2010 small subregions census, and 62 cases in small subregions with zero general households from the 2005 or 2010 census, were excluded from the study. As a result, 79,816 patients were finally included (12,981 stomach, 14,369 colorectal, 9,292 lung, 6,940 female breast, and 1,596 cervical cancer cases) (Figure 1).

### Calculation of ADI and its linkage to patient information

We utilized ADI based on Nakaya’s study,^4^^,^^18^ which was calculated from the data collected by small subregional censuses as follows:

*ADI_i_ = k × (2.99 × proportion of old couple households_i_ + 7.57 × proportion of old single households_i_ + 17.4 × proportion of single-mother households_i_ + 2.22 × proportion of rented houses_i_ + 4.03 × proportion of sales and service workers_i_ + 6.05 × proportion of agricultural workers_i_ + 5.38 × proportion of blue-collar workers_i_ + 18.3 × unemployment rate_i_),*

where *i* is the area index and *k* refers to a positive constant. The large weights in the above equation indicate that the census variables are most likely related to poverty. Gordon proposes the deprivation index as a synthetic estimate of the percentage of poor households in a census area by calculating the value of *k* to match the national mean of the ADI weighted by the number of households in census areas with the national poverty rate.^19^ The value of *k* became 0.01575 based on the population census of 2000 and the estimate of the national poor household rate (8.62%), defined by poor income and subjective low social-class identification, from representative nationwide social survey data, the Japanese General Society Survey (JGSS) cumulative data 2000–2003, using Gordon’s procedure.

We calculated the ADI of each small subregion in Miyagi Prefecture from the 2005 and 2010 small subregion census.^20^^,^^21^ wherein the average value for both years was used as the ADI of that region. We then added the ADI information of the patient’s residence to the data using the small subregional census address codes from the Miyagi Prefectural Cancer Registry based on the diagnostic address information of the Cancer Registry. The mean of the ADI, which corresponds to a synthetic estimate of the percentage of poor households as weighted by the study population, was 9.25%, and the 20th, 40th, 60th, and 80th percentile values were 8.14%, 8.96%, 9.58%, and 10.31%, respectively.

### Statistical analysis

Using the world standard population as the standard population, the age-adjusted incidence of all sites (International Classification of Diseases, 10th revision [ICD-10] codes C00–C96, D00–D09), stomach (C16), colorectal (C18–C20, D01.0–D01.2), lung (C33–C34, D02.1–D02.2), breast (C50, D05), and cervical (C53, D06) cancers by sex was calculated using the ADI quintiles, as weighted by the study population. These five sites of malignancy were recommended for screening by the municipalities, as per the national guidelines. The population was calculated using interpolation from the 2005 and 2010 small subregions census by sex and the 5-year age groups.^20^^,^^21^ Age-adjusted incidence rates included patients with carcinoma in situ, whereas values for invasive cancer of all sites, colorectum, breast, and cervix were also calculated. The same method was used to calculate the incidence of advanced cancer at the target site. The population-based cancer registry classifies the extent of the disease as follows: carcinoma in situ, localized, regional lymph node metastasis, adjacent organ invasion, or distant metastasis. Among these, cases categorized as regional lymph node metastasis, adjacent organ invasion, or distant metastasis were classified as advanced cancer. Moreover, death certificate only (DCO) cases registered only with information from the death certificate and death certificate notification (DCN) cases that were known for the first time from the information on the death certificate with an unknown extent of the disease even after referral to the medical institution were also classified as advanced cancers.

In addition, we examined the association between ADI and cancer incidence using Poisson regression analysis. The relative risk (RR) and 95% confidence intervals (CIs) of other groups were determined in the least deprived areas (1st quintile) as a reference group, and a test for trend was conducted. In doing so, the expected number of cases was calculated from the age-specific incidence rates for the entire study area. We then examined the association between ADI and the proportion of screen-detected cancers using logistic regression analysis adjusted for age. DCO cases were excluded from this analysis because of the absence of information in all cases, and screen-detected cases included those who were detected by medical check-ups in addition to population-based cancer screening. *P* values of less than 0.05 were considered statistically significant. All statistical analyses were performed using Stata (version 14.2; StataCorp, College Station, TX, USA).

### Ethical considerations

This study was approved by the Medical Research Ethics Review Committee of Kanazawa Medical University (reference number: I130).

## RESULTS

Table 1 shows the characteristics of the included patients according to sex and ADI quintile. For both sexes, the proportion of the elderly tended to be high as the deprivation level of the region increased. Among men, the most common type of cancer was colorectum in the least deprived areas and stomach in other areas, whereas women had the highest number of breast cancer cases at all levels. We also observed that a generally higher ADI was attributed to a higher proportion of advanced cancers. Additionally, the proportion of screen-detected cases in the least deprived areas was the highest in all sites for men and in the lungs and breasts for women. Furthermore, a greater difference in the proportion of screen-detected cases between groups was observed in men than in women.

**Table 1.** Characteristics of included patients

Men										

	Deprivation group									

	Q1		Q2		Q3		Q4		Q5	
	(least deprived)								(most deprived)	

**Total population^a^**	199,209		198,183		198,047		199,278		196,238	
**Population by age group^a^**										
0–39 years	102,151	(51.3)	92,782	(46.8)	89,349	(45.1)	90,883	(45.6)	89,225	(45.5)
40–49 years	29,939	(15.0)	25,192	(12.7)	24,584	(12.4)	24,806	(12.4)	23,913	(12.2)
50–59 years	30,015	(15.1)	29,401	(14.8)	30,049	(15.2)	29,412	(14.8)	28,118	(14.3)
60–69 years	21,002	(10.5)	24,322	(12.3)	25,402	(12.8)	25,966	(13.0)	26,280	(13.4)
70–79 years	11,253	(5.6)	17,993	(9.1)	19,357	(9.8)	19,640	(9.9)	20,213	(10.3)
≥80 years	4,848	(2.4)	8,492	(4.3)	9,307	(4.7)	8,571	(4.3)	8,488	(4.3)
**Number of cases (included carcinoma in situ)**										
All sites	6,743		9,239		9,956		10,072		10,238	
Stomach	1,271		1,839		1,966		1,926		1,954	
Colorectum	1,327		1,683		1,772		1,749		1,794	
Lung	858		1,270		1,372		1,493		1,551	
**Number of cases (invasive cancer only)**										
All sites	6,175		8,596		9,272		9,426		9,573	
Colorectum	961		1,252		1,351		1,343		1,382	
**Extent of disease (%)**										
All sites^b^										
Early stage	3,118	(47.1)	4,005	(44.2)	4,336	(44.3)	4,281	(43.2)	4,249	(42.3)
Advanced stage	2,635	(39.8)	3,832	(42.3)	4,199	(42.9)	4,280	(43.2)	4,501	(44.8)
Unknown	872	(13.2)	1,227	(13.5)	1,255	(12.8)	1,341	(13.5)	1,297	(12.9)
Stomach										
Early stage	775	(61.0)	1,040	(56.6)	1,099	(55.9)	1,098	(57.0)	1,049	(53.7)
Advanced stage	412	(32.4)	664	(36.1)	746	(37.9)	682	(35.4)	768	(39.3)
Unknown	84	(6.6)	135	(7.3)	121	(6.2)	146	(7.6)	137	(7.0)
Colorectum										
Early stage	813	(61.3)	1,017	(60.4)	1,016	(57.3)	990	(56.6)	998	(55.6)
Advanced stage	420	(31.7)	575	(34.2)	642	(36.2)	652	(37.3)	685	(38.2)
Unknown	94	(7.1)	91	(5.4)	114	(6.4)	107	(6.1)	111	(6.2)
Lung										
Early stage	216	(25.2)	276	(21.7)	313	(22.8)	322	(21.6)	308	(19.9)
Advanced stage	549	(64.0)	875	(68.9)	915	(66.7)	1,009	(67.6)	1,080	(69.6)
Unknown	93	(10.8)	119	(9.4)	144	(10.5)	162	(10.9)	163	(10.5)
**Screen-detected cases (%)**										
Stomach	39.4		33.9		32.0		32.0		29.9	
Colorectum	35.2		31.7		27.8		30.6		28.4	
Lung	25.1		21.8		23.8		22.2		19.7	



Women										

	Deprivation group									

	Q1		Q2		Q3		Q4		Q5	
	(least deprived)								(most deprived)	

**Total population^a^**	208,927		210,086		209,902		208,984		211,477	
**Population by age group^a^**										
0–39 years	102,449	(49.0)	89,792	(42.7)	86,320	(41.1)	86,189	(41.2)	85,585	(40.5)
40–49 years	30,998	(14.8)	25,366	(12.1)	24,299	(11.6)	24,173	(11.6)	24,409	(11.5)
50–59 years	30,643	(14.7)	29,366	(14.0)	29,940	(14.3)	29,202	(14.0)	29,189	(13.8)
60–69 years	20,862	(10.0)	25,821	(12.3)	26,759	(12.7)	27,696	(13.3)	29,152	(13.8)
70–79 years	14,070	(6.7)	22,898	(10.9)	24,979	(11.9)	24,774	(11.9)	26,239	(12.4)
≥80 years	9,905	(4.7)	16,842	(8.0)	17,603	(8.4)	16,949	(8.1)	16,903	(8.0)
**Number of cases (included carcinoma in situ)**										
All sites	5,476		6,713		6,972		7,065		7,342	
Stomach	619		813		844		879		870	
Colorectum	904		1,258		1,267		1,309		1,306	
Lung	418		494		554		602		680	
Breast	1,425		1,372		1,354		1,385		1,404	
Cervix	304		290		321		307		374	
**Number of cases (invasive cancer only)**										
All sites	4,843		6,033		6,322		6,395		6,673	
Colorectum	757		1,029		1,062		1,103		1,128	
Breast	1,189		1,160		1,172		1,203		1,228	
Cervix	121		126		139		138		176	
**Extent of disease (%)**										
All sites^b^										
Early stage	2,652	(49.4)	3,010	(45.8)	3,150	(46.2)	3,063	(44.3)	3,117	(43.5)
Advanced stage	2,215	(41.3)	2,897	(44.1)	2,997	(44.0)	3,125	(45.2)	3,361	(46.9)
Unknown	500	(9.3)	663	(10.1)	665	(9.8)	727	(10.5)	695	(9.7)
Stomach										
Early stage	314	(50.7)	445	(54.7)	454	(53.8)	421	(47.9)	413	(47.5)
Advanced stage	259	(41.8)	310	(38.1)	330	(39.1)	382	(43.5)	385	(44.3)
Unknown	46	(7.4)	58	(7.1)	60	(7.1)	76	(8.6)	72	(8.3)
Colorectum										
Early stage	431	(47.7)	591	(47.0)	601	(47.4)	627	(47.9)	614	(47.0)
Advanced stage	408	(45.1)	591	(47.0)	573	(45.2)	600	(45.8)	624	(47.8)
Unknown	65	(7.2)	76	(6.0)	93	(7.3)	82	(6.3)	68	(5.2)
Lung										
Early stage	160	(38.3)	163	(33.0)	183	(33.0)	173	(28.7)	216	(31.8)
Advanced stage	237	(56.7)	291	(58.9)	321	(57.9)	368	(61.1)	394	(57.9)
Unknown	21	(5.0)	40	(8.1)	50	(9.0)	61	(10.1)	70	(10.3)
Breast										
Early stage	942	(66.1)	883	(64.4)	895	(66.1)	868	(62.7)	842	(60.0)
Advanced stage	412	(28.9)	401	(29.2)	387	(28.6)	436	(31.5)	491	(35.0)
Unknown	71	(5.0)	88	(6.4)	72	(5.3)	81	(5.8)	71	(5.1)
Cervix										
Early stage	231	(76.0)	222	(76.6)	229	(71.3)	220	(71.7)	276	(73.8)
Advanced stage	57	(18.8)	57	(19.7)	75	(23.4)	70	(22.8)	82	(21.9)
Unknown	16	(5.3)	11	(3.8)	17	(5.3)	17	(5.5)	16	(4.3)
**Screen-detected cases (%)**										
Stomach	26.4		28.2		25.6		24.1		26.3	
Colorectum	25.1		25.1		24.5		25.2		23.3	
Lung	32.2		31.0		31.3		27.5		26.6	
Breast	34.3		34.1		33.4		30.8		31.9	
Cervix	39.6		40.8		44.0		39.0		41.2	


^a^Average of 2005 and 2010.

^b^Excluding leukemia and multiple myeloma.

Table 2 shows the age-adjusted incidence by sex and ADI quintile and the relative risk with reference to the least deprived areas (1st quintile). In cases including carcinoma in situ, the relative risk tended to increase significantly as the ADI increased for all sites and lung cancers in men and for lung and cervical cancers in women. A significant increase in the relative risk was also observed in the most deprived areas (5^th^ quintile) for all sites and lung cancers in men and for cervical cancers in women by 6%, 20%, and 29%, respectively. Moreover, the age-adjusted incidence and relative risk of all sites in men and cervical cancer showed a similar trend in invasive cancer. On the other hand, breast cancers showed a significant decrease in relative risk with increasing deprivation index in cases, including carcinoma in situ and in other groups relative to the least deprived areas (RR = 0.888 in the most deprived areas). In invasive cancer, the trend towards a lower relative risk was not statistically significant, although the relative risk in the most deprived areas was 0.921 (95% CI, 0.851–0.998), indicating a significant decrease.

**Table 2.** Association between areal deprivation index and cancer incidence

	Deprivation group	Men		Women	
	
		Age-adjusted incidence rate (per 100,000)	Relative risk (95% CI)	Age-adjusted incidence rate (per 100,000)	Relative risk (95% CI)
Included carcinoma in situ					
All sites	Q1(least)	328.9	1.000	246.7	1.000
	Q2	332.3	1.013 (0.982–1.046)	242.7	0.967 (0.934–1.003)
	Q3	336.7	1.029 (0.997–1.061)	240.9	0.967 (0.934–1.002)
	Q4	343.3	1.048 (1.017–1.081)	243.2	0.990 (0.956–1.025)
	Q5(most)	342.9	1.058 (1.026–1.091)	250.3	1.015 (0.969–1.039)
	*P* for trend		<0.001		0.295

Stomach	Q1(least)	61.2	1.000	23.9	1.000
	Q2	65.0	1.069 (0.996–1.149)	22.6	0.933 (0.840–1.036)
	Q3	65.8	1.077 (1.003–1.155)	21.6	0.921 (0.830–1.022)
	Q4	65.1	1.062 (0.989–1.140)	23.1	0.971 (0.876–1.077)
	Q5(most)	64.9	1.069 (0.996–1.147)	21.8	0.935 (0.843–1.036)
	*P* for trend		0.178		0.523

Colorectum	Q1(least)	65.3	1.000	35.0	1.000
	Q2	62.7	0.968 (0.901–1.040)	36.1	1.004 (0.922–1.093)
	Q3	62.2	0.963 (0.897–1.034)	34.8	0.962 (0.884–1.048)
	Q4	62.3	0.956 (0.890–1.026)	36.3	1.005 (0.924–1.094)
	Q5(most)	63.2	0.975 (0.908–1.047)	34.5	0.974 (0.895–1.061)
	*P* for trend		0.501		0.600

Lung	Q1(least)	39.6	1.000	15.8	1.000
	Q2	41.8	1.043 (0.956–1.137)	14.2	0.842 (0.739–0.959)
	Q3	41.8	1.055 (0.968–1.149)	14.0	0.898 (0.791–1.019)
	Q4	44.9	1.164 (1.071–1.266)	15.7	0.985 (0.869–1.116)
	Q5(most)	47.7	1.200 (1.104–1.305)	18.5	1.081 (0.957–1.221)
	*P* for trend		<0.001		0.004

Breast	Q1(least)			74.7	1.00
	Q2			69.8	0.900 (0.835–0.969)
	Q3			65.6	0.872 (0.809–0.939)
	Q4			66.9	0.896 (0.832–0.965)
	Q5(most)			66.4	0.888 (0.825–0.956)
	*P* for trend				0.005

Cervix	Q1(least)			18.3	1.00
	Q2			19.0	0.989 (0.842–1.161)
	Q3			20.6	1.107 (0.946–1.295)
	Q4			20.1	1.060 (0.905–1.243)
	Q5(most)			26.0	1.289 (1.108–1.500)
	*P* for trend				0.001

Invasive cancer only					
All sites	Q1(least)	300.7	1.000	212.7	1.000
	Q2	307.1	1.026 (0.993–1.060)	208.5	0.967 (0.931–1.004)
	Q3	312.3	1.042 (1.009–1.076)	208.3	0.974 (0.938–1.001)
	Q4	319.5	1.068 (1.034–1.103)	211.0	0.995 (0.959–1.033)
	Q5(most)	319.2	1.077 (1.043–1.112)	217.6	1.013 (0.976–1.051)
	*P* for trend		<0.001		0.112

Colorectum	Q1(least)	46.9	1.000	28.3	1.000
	Q2	45.6	0.983 (0.904–1.070)	28.1	0.969 (0.882–1.064)
	Q3	46.4	1.002 (0.922–1.088)	27.8	0.952 (0.867–1.045)
	Q4	46.6	1.003 (0.923–1.090)	29.4	1.001 (0.913–1.098)
	Q5(most)	48.5	1.027 (0.946–1.116)	29.3	0.996 (0.909–1.093)
	*P* for trend		0.374		0.683

Breast	Q1(least)			61.7	1.000
	Q2			57.9	0.903 (0.833–0.980)
	Q3			55.4	0.895 (0.825–0.970)
	Q4			57.0	0.923 (0.852–1.000)
	Q5(most)			57.4	0.921 (0.851–0.998)
	*P* for trend				0.130

Cervix	Q1(least)			6.5	1.000
	Q2			7.1	1.007 (0.785–1.293)
	Q3			7.3	1.106 (0.867–1.411)
	Q4			7.3	1.104 (0.865–1.409)
	Q5(most)			10.3	1.393 (1.105–1.756)
	*P* for trend				0.003

CI, confidence interval.

Table 3 shows the age-adjusted incidence rates by sex and ADI quintile and the relative risk with reference to the least deprived areas for advanced cancer. We observed a trend toward a significantly higher relative risk for all sites, stomach, colorectal, and lung cancers in deprived areas, and a significant increase in the relative risk of the most deprived areas in men by 17% (colorectum) to 32% (lung). Similarly, the incidence of all sites and lung cancer indicated a significant upward tendency among women as ADI increased (*P* < 0.001 and *P* = 0.011, respectively), and cervical cancer incidence tended to be higher in deprived areas (*P* = 0.058).

**Table 3.** Association between areal deprivation index and advanced cancer incidence

	Deprivation group	Men		Women	
	
		Age-adjusted incidence rate (per 100,000)	Relative risk (95% CI)	Age-adjusted incidence rate (per 100,000)	Relative risk (95% CI)
All sites^a^	Q1(least)	125.8	1.000	89.6	1.000
	Q2	132.7	1.062 (1.011–1.116)	91.8	0.987 (0.934–1.043)
	Q3	137.1	1.094 (1.042–1.149)	88.8	0.980 (0.928–1.035)
	Q4	142.9	1.129 (1.076–1.186)	96.6	1.035 (0.980–1.093)
	Q5(most)	148.2	1.182 (1.126–1.240)	102.7	1.089 (1.032–1.149)
	*P* for trend		<0.001		<0.001

Stomach	Q1(least)	19.3	1.000	9.1	1.000
	Q2	23.2	1.177 (1.041–1.331)	7.9	0.839 (0.711–0.989)
	Q3	24.3	1.243 (1.102–1.402)	7.7	0.853 (0.725–1.003)
	Q4	23.0	1.151 (1.019–1.301)	9.9	1.003 (0.857–1.174)
	Q5(most)	25.3	1.290 (1.144–1.454)	9.3	0.991 (0.846–1.160)
	*P* for trend		0.001		0.199

Colorectum	Q1(least)	20.4	1.000	14.3	1.000
	Q2	20.5	1.031 (0.909–1.169)	15.1	1.017 (0.897–1.154)
	Q3	22.0	1.086 (0.960–1.228)	14.2	0.940 (0.828–1.067)
	Q4	22.8	1.114 (0.985–1.259)	15.4	0.997 (0.879–1.131)
	Q5(most)	23.9	1.166 (1.033–1.317)	16.0	1.012 (0.893–1.147)
	*P* for trend		0.004		0.929

Lung	Q1(least)	25.5	1.000	8.1	1.000
	Q2	29.1	1.128 (1.014–1.255)	7.9	0.856 (0.721–1.017)
	Q3	28.1	1.105 (0.994–1.228)	7.5	0.901 (0.762–1.065)
	Q4	30.5	1.238 (1.115–1.373)	9.0	1.046 (0.888–1.231)
	Q5(most)	33.9	1.316 (1.188–1.459)	10.2	1.094 (0.931–1.285)
	*P* for trend		<0.001		0.011

Breast	Q1(least)			20.8	1.000
	Q2			20.3	0.903 (0.787–1.036)
	Q3			18.2	0.855 (0.744–0.982)
	Q4			21.0	0.969 (0.847–1.109)
	Q5(most)			23.1	1.070 (0.938–1.219)
	*P* for trend				0.133

Cervix	Q1(least)			2.7	1.000
	Q2			3.0	0.922 (0.639–1.332)
	Q3			3.5	1.197 (0.849–1.690)
	Q4			3.3	1.126 (0.794–1.597)
	Q5(most)			4.3	1.301 (0.928–1.824)
	*P* for trend				0.058

CI, confidence interval.

^a^Excluding leukemia and multiple myeloma.

Table 4 shows the logistic regression analysis results of the association between ADI and the proportion of screen-detected cases after adjusting for age by sex. In men, cancers tend to be detected less likely by screening in deprived areas, with odds ratios in the most deprived areas of 0.726 (95% CI, 0.621–0.849) and 0.794 (95% CI, 0.677–0.931) for stomach and colorectal cancers, respectively. This showed that the proportion of cases detected by screening was significantly reduced. Meanwhile, women tended to have lower proportions of screen-detected lung cancers in deprived areas (*P* = 0.053), which was similar to the proportions in men.

**Table 4.** Associations between areal deprivation index and proportion of cases detected by screening

	Deprivation group	Men			Women		
	
		Number of cases	Number of screen-detected cases	Odds ratio (95% CI)	Number of cases	Number of screen-detected cases	Odds ratio (95% CI)
Stomach	Q1(least)	1,201	473	1.000	545	144	1.000
	Q2	1,716	582	0.868 (0.743–1.015)	731	206	1.233 (0.953–1.595)
	Q3	1,823	583	0.792 (0.678–0.924)	759	194	1.076 (0.830–1.394)
	Q4	1,791	574	0.795 (0.681–0.929)	783	189	0.999 (0.771–1.295)
	Q5(most)	1,799	538	0.726 (0.621–0.849)	754	198	1.095 (0.846–1.418)
	*P* for trend			<0.001			0.791

Colorectum	Q1(least)	1,270	447	1.000	837	210	1.000
	Q2	1,592	504	0.921 (0.786–1.080)	1,145	287	1.045 (0.846–1.290)
	Q3	1,683	468	0.777 (0.662–0.911)	1,146	281	1.020 (0.826–1.261)
	Q4	1,660	508	0.876 (0.748–1.026)	1,212	306	1.033 (0.839–1.273)
	Q5(most)	1,693	481	0.794 (0.677–0.931)	1,185	276	0.952 (0.770–1.176)
	*P* for trend			0.006			0.584

Lung	Q1(least)	756	190	1.000	351	113	1.000
	Q2	1,103	241	0.901 (0.722–1.124)	422	131	1.005 (0.734–1.377)
	Q3	1,218	290	1.032 (0.832–1.279)	453	142	1.096 (0.803–1.496)
	Q4	1,286	285	0.942 (0.760–1.167)	495	136	0.888 (0.652–1.210)
	Q5(most)	1,369	270	0.804 (0.649–0.997)	582	155	0.792 (0.587–1.068)
	*P* for trend			0.079			0.053

Breast	Q1(least)				1,398	480	1.000
	Q2				1,343	458	1.027 (0.875–1.205)
	Q3				1,322	441	1.013 (0.861–1.190)
	Q4				1,352	416	0.890 (0.757–1.047)
	Q5(most)				1,374	438	0.933 (0.794–1.096)
	*P* for trend						0.132

Cervix	Q1(least)				298	118	1.000
	Q2				287	117	1.096 (0.779–1.542)
	Q3				316	139	1.357 (0.971–1.896)
	Q4				305	119	1.076 (0.767–1.509)
	Q5(most)				369	152	1.095 (0.794–1.509)
	*P* for trend						0.713

CI, confidence interval.

## DISCUSSION

This study examined the association between ADI and cancer incidence and the proportion of screen-detected cancer by the site. The results showed that lung and cervical cancers in both sexes tended to increase in the deprived areas. In addition, for advanced cancers, stomach and colorectal cancers also tended to increase in deprived areas among men. Moreover, regarding the association between ADI and the proportion of cancer detected by screening, the proportion of screen-detected cancer tended to generally decrease in the group with a high ADI among men, a significant association was observed in stomach and colorectal cancers. In contrast, no significant differences were observed in women.

The socioeconomic gradient in lung cancer incidence is consistent with many previous western studies,^8^^,^^9^^,^^22^^–^^24^ which may be explained by the differences in smoking prevalence, since this is a major risk factor for lung cancer. In particular, high smoking rates have been reported among people with low education, low occupational status, and low income.^2^^,^^25^^–^^27^ Furthermore, in individual-level studies in Japan, the ADI used in this study showed a significant positive correlation with the current smoking rate.^7^

Our results of a socioeconomic gradient in cervical cancer incidence were consistent with those of previous studies.^2^^,^^8^^–^^10^ HPV infection is a risk factor for cervical cancer, and a higher prevalence of high-risk HPV infection, particularly among poor women in the United States National Health and Nutrition Examination Survey,^28^ and among those living in deprived areas with elevated ADI values in a national survey in England^29^ have been reported. Smoking is also a risk factor for cervical cancer, wherein a higher smoking prevalence in lower socioeconomic strata may increase the incidence of cervical cancer. Additionally, the early detection and treatment of precancerous lesions by cervical cancer screening can prevent cancer progression. There are reports that more deprived areas have lower rates of cervical cancer screening,^30^ and the disparity in screening uptake may affect cancer incidence rates. HPV vaccination has been recommended to eliminate cervical cancer. HPV vaccination in Japan lags behind that in other countries and should be promoted to reduce poverty disparities.

Breast cancer incidence in women showed a significant increase in affluent areas with low levels of deprivation, including carcinoma in situ, while the association between the two was weakened in cases of invasive cancer alone. Furthermore, the incidence of advanced cancer was reported to have increased, albeit not significantly, in the most deprived areas. These results indicated that the proportion of breast cancer cases detected at an early stage was higher in affluent areas, whereas the proportion of breast cancer cases detected by screening was slightly lower in the deprived areas, but the difference was not statistically significant. In addition to screening behavior, it is possible that the incidence of advanced cancer is rising because residents from deprived areas often have delayed consultation behavior in cases with symptom onset. The increase in the incidence of breast carcinoma in situ and invasive cancers in affluent areas with low deprivation indicators among women is consistent with the results of previous studies.^2^ These studies attributed this increase to the high proportion of nulliparous or elderly primiparous women in these areas, both of which have an increased risk of breast cancer.

The present study also showed that the incidence of advanced cancer in men tended to increase significantly in the group with high deprivation indicators for stomach, colon, and lung cancers. As shown in Table 4, the proportion of stomach and colorectal cancer cases detected by screening in men was significantly lower in the group with high ADI values, and the difference in the screening status of each group may affect the incidence of advanced cancer. Previous western studies have shown that residents in deprived areas have a high proportion of people who have not been screened for cancer^30^ and a high proportion of advanced cancer cases at the time of diagnosis.^14^^,^^15^ Furthermore, a Japanese study examining the relationship between medical insurance type and cancer screening rate found that the cancer screening rate among municipal national health subscribers who were not as wealthy as employee insurance subscribers was generally lower than that of other insurance subscribers.^31^^,^^32^

Regarding areal deprivation and the proportion of cancer cases detected by screening, men tended to have a lower proportion of screen-detected cancer at any site in more deprived areas; however, this trend was not observed in women, except for lung cancer. In Japan, cancer screening is provided by local governments or workplaces; thus, there are more opportunities for regular employees to undergo cancer screening compared to non-regular workers. However, the proportion of regular female employees in Japan is lower than that of male employees even among highly educated populations^33^; therefore, the difference in the proportion of regular employees among social classes is considered to be smaller in women than in men. This likely contributed to the association between ADI values and the proportion of screen-detected cancer in many sites that was not observed in women.

Only a few studies have examined the association between areal deprivation and cancer incidence and mortality in Japan compared to other countries. However, such studies using population-based cancer registry data from the Osaka Prefecture have found a significant increase in advanced cancer incidence in areas with the highest deprivation levels for stomach, colorectal, lung, breast, and cervical cancers in both sexes compared to areas with the lowest deprivation levels.^16^ Additionally, studies examining the association between ADI calculated on a municipal basis and cancer mortality across Japan also found higher mortality rates at higher deprivation levels in 2010–2014 for 10 common sites and malignant mesothelioma in men and several sites, including colorectum, lung, and cervix, in women.^34^ In contrast, studies examining the association between areal deprivation and cancer incidence in cohort studies have not found a significant association between ADI and the incidence of total and major cancers.^17^ However, since this study examined the impact of areal deprivation on cancer incidence via individual and community characteristics after adjusting for individuals’ smoking, alcohol use, body mass index, and exercise habits, a simple comparison with the results of this study and the present study cannot be made.

Poverty areas with high ADI generally tended to have higher cancer incidence rates, both in our study and in the previous study mentioned above.^16^ This may be attributed not only to individual factors, such as socioeconomic disadvantage, with many residents being poor, but also to area factors, such as poor living conditions. Area factors include availability of health-promoting resources and environments, such as grocery stores and playgrounds, that affect individual health behavior, and they may be linked to health disparities in which access to them has a tendency to be more difficult for residents in deprived areas.^4^^–^^7^

The strength of this study was the evaluation of the association between socioeconomic status and cancer incidence in Japan using population-based cancer registry data. Using incidence rather than mortality as an index, it was possible to examine the effects of socioeconomic conditions through risk factors and disease screening by removing the effects on prognosis. Moreover, Miyagi Prefecture has maintained high cancer screening uptake rates at all targeted sites in Japan, ranging from 33.8% (lung) to 46.2% (female breast), based on the Comprehensive Survey of Living Conditions 2010^35^; therefore, it was suitable for examining the impact of socioeconomic conditions on cancer screening.

This study has some limitations. First, this study was conducted in only one prefecture in Japan. Therefore, the statistical power was not necessarily adequate for a cancer site with a small number of cases. Second, we excluded 12.6% of the patients living in areas with border changes in the 2005 and 2010 small subregions census, which could have affected the results. Lastly, DCO and DCN cases with unknown disease extents were classified as advanced cancers in the analysis, and 11.8% of all cases had an unknown extent of disease. Thus, considering an association between ADI and the proportion of misclassification of disease extent in DCO and DCN cases and the proportion of advanced cancers in cases with an unknown extent of disease, the study results may change. However, the results did not change substantially in the analysis, when DCO and DCN were excluded from advanced cancer.

### Conclusion

We have shown that regional socioeconomic disparities in Japan may affect the incidence and early diagnosis of cancer. Specifically, we found that the incidence of lung and cervical cancers was higher in deprived areas with high ADI values for both sexes, and the incidence of advanced cancers was higher in the stomach, colorectum, and lungs in men and in the lungs in women. Moreover, the proportion of stomach and colorectal cancers detected by screening in men tended to be significantly lower in deprived areas. Differences may have influenced the results in the distribution of risk factors, including smoking and HPV infection, and the rate of screening visits. Therefore, to prevent cancer in Japan, it is necessary to take measures against tobacco use and HPV infection and promote early medical treatment among poor people. Additionally, to improve the cancer screening rate in Japan, it is important to eliminate the disparity between men and women and make cancer screening accessible for non-regular male employees.

## References

[1] Boscoe FP, Johnson CJ, Sherman RL, Stinchcomb DG, Lin G, Henry KA. The relationship between area poverty rate and site-specific cancer incidence in the United States. Cancer. 2014;120(14):2191–2198. 10.1002/cncr.28632 24866103 PMC4232004

[2] Mihor A, Tomsic S, Zagar T, Lokar K, Zadnik V. Socioeconomic inequalities in cancer incidence in Europe: a comprehensive review of population-based epidemiological studies. Radiol Oncol. 2020;54(1):1–13. 10.2478/raon-2020-0008 32074075 PMC7087422

[3] Ministry of Housing CLG. The English Indices of Deprivation 2019 (IoD2019). 2019. Accessed June 20, 2020. https://assets.publishing.service.gov.uk/government/uploads/system/uploads/attachment_data/file/835115/IoD2019_Statistical_Release.pdf.

[4] Nakaya T, Honjo K, Hanibuchi T, ; Japan Public Health Center-based Prospective Study Group. Associations of all-cause mortality with census-based neighbourhood deprivation and population density in Japan: a multilevel survival analysis. PLoS One. 2014;9(6):e97802. 10.1371/journal.pone.0097802 24905731 PMC4048169

[5] Gomez SL, Shariff-Marco S, DeRouen M, . The impact of neighborhood social and built environment factors across the cancer continuum: current research, methodological considerations, and future directions. Cancer. 2015;121(14):2314–2330. 10.1002/cncr.29345 25847484 PMC4490083

[6] Menvielle G, Kulhánová I, Bryère J, . Tobacco-attributable burden of cancer according to socioeconomic position in France. Int J Cancer. 2018;143(3):478–485. 10.1002/ijc.31328 29457849

[7] Hanibuchi T, Nakaya T. Associations of neighborhood socioeconomic conditions with self-rated health, mental distress, and health behaviors: a nationwide cross-sectional study in Japan. Prev Med Rep. 2020;18:101075. 10.1016/j.pmedr.2020.101075 32181123 PMC7063227

[8] Hoebel J, Kroll LE, Fiebig J, . Socioeconomic inequalities in total and site-specific cancer incidence in Germany: a population-based registry study. Front Oncol. 2018;8:402. 10.3389/fonc.2018.00402 30319967 PMC6167637

[9] Singh GK, Jemal A. Socioeconomic and racial/ethnic disparities in cancer mortality, incidence, and survival in the United States, 1950–2014: over six decades of changing patterns and widening inequalities. J Environ Public Health. 2017;2017:2819372. 10.1155/2017/2819372 28408935 PMC5376950

[10] Singh GK, Miller BA, Hankey BF, Edwards BK. Persistent area socioeconomic disparities in U.S. incidence of cervical cancer, mortality, stage, and survival, 1975–2000. Cancer. 2004;101(5):1051–1057. 10.1002/cncr.20467 15329915

[11] Wilson LF, Green AC, Jordan SJ, Neale RE, Webb PM, Whiteman DC. The proportion of cancers attributable to social deprivation: a population-based analysis of Australian health data. Cancer Epidemiol. 2020;67:101742. 10.1016/j.canep.2020.101742 32512495

[12] Smith D, Thomson K, Bambra C, Todd A. The breast cancer paradox: a systematic review of the association between area-level deprivation and breast cancer screening uptake in Europe. Cancer Epidemiol. 2019;60:77–85. 10.1016/j.canep.2019.03.008 30927689 PMC6547165

[13] Lundqvist A, Andersson E, Ahlberg I, Nilbert M, Gerdtham U. Socioeconomic inequalities in breast cancer incidence and mortality in Europe-a systematic review and meta-analysis. Eur J Public Health. 2016;26(5):804–813. 10.1093/eurpub/ckw070 27221607 PMC5054273

[14] Boscoe FP, Henry KA, Sherman RL, Johnson CJ. The relationship between cancer incidence, stage and poverty in the United States. Int J Cancer. 2016;139(3):607–612. 10.1002/ijc.30087 26991033

[15] Henry KA, Sherman RL, McDonald K, . Associations of census-tract poverty with subsite-specific colorectal cancer incidence rates and stage of disease at diagnosis in the United States. J Cancer Epidemiol. 2014;2014:823484. 10.1155/2014/823484 25165475 PMC4137551

[16] Ito Y. Socioeconomic inequalities in cancer outcome in Japan. Gan To Kagaku Ryoho. 2020;47(7):1007–1011 [in Japanese]. 32668840

[17] Miki Y, Inoue M, Ikeda A, ; JPHC Study Group. Neighborhood deprivation and risk of cancer incidence, mortality and survival: results from a population-based cohort study in Japan. PLoS One. 2014;9(9):e106729. 10.1371/journal.pone.0106729 25184297 PMC4153661

[18] Ito Y, Nakaya T, Nakayama T, . Socioeconomic inequalities in cancer survival: a population-based study of adult patients diagnosed in Osaka, Japan, during the period 1993–2004. Acta Oncol. 2014;53(10):1423–1433. 10.3109/0284186X.2014.912350 24865119

[19] Gordon D. Census based deprivation indices: their weighting and validation. J Epidemiol Community Health. 1995;49(Suppl 2):S39–S44. 10.1136/jech.49.Suppl_2.S39 8594133 PMC1060875

[20] Statistics Bureau of Japan. 2005 Population Census. Tabulation for Small Areas. Accessed April 30, 2022. https://www.e-stat.go.jp/stat-search/files?page=2&toukei=00200521&tstat=000001007251 [in Japanese].

[21] Statistics Bureau of Japan. 2010 Population Census. Tabulation for Small Areas. Accessed April 30, 2022. https://www.e-stat.go.jp/stat-search/files?page=1&toukei=00200521&tstat=000001039448 [in Japanese].

[22] Shack L, Jordan C, Thomson CS, Mak V, Møller H; UK Association of Cancer Registries. Variation in incidence of breast, lung and cervical cancer and malignant melanoma of skin by socioeconomic group in England. BMC Cancer. 2008;8:271. 10.1186/1471-2407-8-271 18822122 PMC2577116

[23] Tweed EJ, Allardice GM, McLoone P, Morrison DS. Socio-economic inequalities in the incidence of four common cancers: a population-based registry study. Public Health. Jan 2018;154:1–10. 10.1016/j.puhe.2017.10.005 29128730 PMC5764071

[24] Hajizadeh M, Johnston GM, Manos D. Socio-economic inequalities in lung cancer incidence in Canada, 1992–2010: results from the Canadian Cancer Registry. Public Health. 2020;185:189–195. 10.1016/j.puhe.2020.04.023 32645506

[25] Fukuda Y, Nakao H, Imai H. Different income information as an indicator for health inequality among Japanese adults. J Epidemiol. 2007;17(3):93–99. 10.2188/jea.17.93 17545696 PMC7058454

[26] Tabuchi T, Kondo N. Educational inequalities in smoking among Japanese adults aged 25–94 years: nationally representative sex- and age-specific statistics. J Epidemiol. 2017;27(4):186–192. 10.1016/j.je.2016.05.007 28142048 PMC5376315

[27] Tomioka K, Kurumatani N, Saeki K. The association between education and smoking prevalence, independent of occupation: a nationally representative survey in Japan. J Epidemiol. 2020;30(3):136–142. 10.2188/jea.JE20180195 30828035 PMC7025916

[28] Kahn JA, Lan D, Kahn RS. Sociodemographic factors associated with high-risk human papillomavirus infection. Obstet Gynecol. 2007;110(1):87–95. 10.1097/01.AOG.0000266984.23445.9c 17601901

[29] Tanton C, Soldan K, Beddows S, . High-risk human papillomavirus (HPV) infection and cervical cancer prevention in Britain: evidence of differential uptake of interventions from a probability survey. Cancer Epidemiol Biomarkers Prev. 2015;24(5):842–853. 10.1158/1055-9965.EPI-14-1333 25737331 PMC4435666

[30] Kurani SS, McCoy RG, Lampman MA, . Association of neighborhood measures of social determinants of health with breast, cervical, and colorectal cancer screening rates in the US Midwest. JAMA Netw Open. 2020;3(3):e200618. 10.1001/jamanetworkopen.2020.0618 32150271 PMC7063513

[31] Tabuchi T, Nakayama T, Tsukuma T. Disparities in cancer screening rates in Japan: the impact of medical insurance. Jpn Med J. 2012;4605:84–88 [in Japanese].

[32] Morishima T, Sato A, Nakata K, . Proportion of screening detected and early-stage cancer among cancer patients by types of medical insurance. J Health Welfare Stat. 2020;67(5):1–6 [in Japanese].

[33] Statistics and Information Department, Minister’s Secretariat, Ministry of Health, Labour and Welfare, eds. *Graphical Review of Japanese Household -From Comprehensive Survey of Living Conditions, 2010*. 2012;43. [in Japanese].

[34] Nakaya T, Ito Y, eds. *The Atlas of Health Inequalities in Japan*. Springer; 2020. doi:10.1007/978-3-030-22707-4. 10.1007/978-3-030-22707-4

[35] Statistics and Information Department, Minister’s Secretariat, Ministry of Health, Labour and Welfare, eds. *Comprehensive Survey of Living Conditions 2010*. vol. 4. Health, Labour and Welfare Statistics Association; 2012. [in Japanese].

